# Running away from the jab: factors associated with COVID-19 vaccine hesitancy in Brazil

**DOI:** 10.11606/s1518-8787.2021055003903

**Published:** 2021-11-16

**Authors:** Marco Antonio Catussi Paschoalotto, Eduardo Polena Pacheco Araújo Costa, Sara Valente de Almeida, Joana Cima, Joana Gomes da Costa, João Vasco Santos, Pedro Pita Barros, Claudia Souza Passador, João Luiz Passador

**Affiliations:** I Universidade NOVA de Lisboa Nova School of Business and Economics Carcavelos Portugal Universidade NOVA de Lisboa. Nova School of Business and Economics. Carcavelos, Portugal; II Imperial College London Faculty of Health Sciences London England Imperial College London. Faculty of Health Sciences. London, England; III Universidade do Minho Núcleo de Investigação em Políticas Económicas e Empresariais Braga Portugal Universidade do Minho. Núcleo de Investigação em Políticas Económicas e Empresariais. Braga, Portugal; IV Universidade do Porto Faculdade de Economia e Gestão Porto Portugal Universidade do Porto. Faculdade de Economia e Gestão. Porto, Portugal; V Universidade do Porto Faculdade de Medicina Departamento Medicina da Comunidade Porto Portugal Universidade do Porto. Faculdade de Medicina. MEDCIDS – Departamento Medicina da Comunidade, Informação e Decisão em Saúde. Porto, Portugal; VI Universidade do Porto Faculdade de Medicina Centro de Investigação em Tecnologias e Serviços de Saúde Porto Portugal Universidade do Porto. Faculdade de Medicina. Centro de Investigação em Tecnologias e Serviços de Saúde. Porto, Portugal; VII Unidade de Saúde Pública ACES Grande Porto VIII Vila Nova de Gaia Portugal ARS Norte. ACES Grande Porto VIII - Espinho/Gaia. Unidade de Saúde Pública. Vila Nova de Gaia, Portugal; VIII Universidade de São Paulo Faculdade de Economia, Administração e Contabilidade de Ribeirão Preto Paulo Brasil Universidade de São Paulo. Faculdade de Economia, Administração e Contabilidade de Ribeirão Preto. Ribeirão Preto, São Paulo, Brasil

**Keywords:** COVID-19 Vaccines, Vaccination Refusal, Socioeconomic Factors, Political Activism, Health Knowledge, Attitudes, Practice

## Abstract

**OBJECTIVE:**

To investigate how sociodemographic conditions, political factors, organizational confidence, and non-pharmaceutical interventions compliance affect the COVID-19 vaccine hesitancy in Brazil.

**METHODS:**

Data collection took place between November 25th, 2020 and January 11th, 2021 using a nationwide online survey. Subsequently, the researches performed a descriptive analysis on the main variables and used logistic regression models to investigate the factors associated with COVID-19 vaccine hesitancy.

**RESULTS:**

Less concern over vaccine side effects could improve the willingness to be vaccinated (probability changed by 7.7 pp; p < 0.10). The current vaccine distrust espoused by the Brazilian president is associated with vaccine hesitancy, among his voter base. Lower performance perception (“Very Bad” with 10.7 pp; p < 0.01) or higher political opposition (left-oriented) regarding the current presidency is associated with the willingness to be vaccinated. Higher compliance with non-pharmaceutical interventions (NPIs) is usually positively associated with the willingness to take the COVID-19 vaccine (+1 score to NPI compliance index is associated with higher willingness to be vaccinated by 1.4 pp, p < 0.05).

**CONCLUSION:**

Willingness to be vaccinated is strongly associated with political leaning, perceived federal government performance, vaccine side effects, and compliance with non-pharmaceutical interventions (NPIs).

## INTRODUCTION

By July 2021, the COVID-19 pandemic had already resulted in more than 186 million cases and 4 million deaths worldwide, with Brazil ranking third place in the number of cases and second in the number of deaths^1^. In a global effort to contain the spread of the new virus, countries adopted several non-pharmacological interventions (NPI) such as social distancing^2–4^ and face mask use^5^. But despite the importance of such measures, the solution to the pandemic rests on the success of vaccination programs^6,7^.

After the extraordinary efforts made to rapidly research and develop effective COVID-19 vaccines and their recent rollout, researchers and the media have pointed to a growing concern regarding public confidence in the vaccination process. In fact, “anti-vaccine movements” can foster vaccine hesitancy, reducing the population’s willingness to be vaccinated^6–13^.

Several surveys have been used to characterize behaviours concerning vaccine hesitancy and NPI compliance^10–18^. According to the existing literature, sociodemographic conditions (e.g., education, age, or job occupation)^10,18–20^ and political and organizational trust aspects^5,8–10,12,13^, can affect people’s willingness to be vaccinated.

One of the countries with the highest number of COVID-19 cases and deaths^1^, Brazil has a population of diverse sociodemographic backgrounds^20–23^ and is governed by a president with a long history of questioning scientific findings, including vaccine efficacy and safety^24–26^. Reducing vaccine hesitancy will largely determine Brazil’s – and other low-middle income countries (LMICs) – success in controlling the current pandemic.

Given this context, this study investigates the factors associated with COVID-19 vaccine hesitancy in Brazil. Using a nationwide online survey, we analyse how sociodemographic conditions, political factors, organizational confidence, and non-pharmaceutical interventions compliance influence the population’s willingness to be vaccinated.

## METHODS

This study was approved by the Research Ethics Committee at NOVA School of Business and Economics (Portugal) on November 23^rd^, 2020, via letter sent by the Scientific Council’s president. Regarding Brazilian ethical standards, the research complied with the National Health Council Resolution 466/12^a^. In its first page, the online survey highlighted the research characteristics and information, anonymity assurance, data protection, and a consent form.

### Data

Data collection took place between November 25^th^, 2020 and January 11^th^, 2021, period before the second COVID-19 wave, considered the deadliest so far, and before the first COVID-19 vaccine (Coronavac – Sinovac/Butantã) was introduced. Using an online survey built on Qualtrics software and disseminated on different social networks (Facebook, Instagram, WhatsApp, and email groups), we sought to collect a diversified base of responses from all Brazilian regions and different social sectors.

Table 1 describes the survey data and compares it to the national averages.

**Table 1:** Sample characteristics (Survey Data) x National characteristics (National Data).

Variable			Survey Data		National Data		
States and municipalities (number)^a^						
	States		24		27	
	Capitals		20		27	
	Municipalities		263		5,570	
Gender (%)						
	Male		37.9		48.2	
	Female		61.7		51.8	
	Other/No answer		0.4			
Age (%)						
	≤ 18 years	0.9		< 19 years		33.1
	19 to 25 years	30.6		20 to 24 years		9.0
	26 to 32 years	20.9		25 to 34 years		17.1
	33 to 45 years	27.4		35 to 44 years		14.0
	46 to 64 years	18.4		45 to 64 years		19.2
	65 to 79 years	1.9		65 to 79 years		6.0
	≥ 80 years	0.1		≥ 80 years		1.6
Education (%)						
	Elementary school		0.5		55.8	
	High school		14.4		30.1	
	University – Bachelor		40.5		14.1	
	University – MBAs and specializations		20.8			
	University – Master’s		14.1			
	University – Doctorate		9.7			
Residence area (%)						
	Urban		97.4		84.4	
	Rural		2.6		15.6	
Households (%; average number)						
	One/Live alone		7.9		30.9	
	Two		33.3			
	Three		26.9		30.4	
	Four		20.7		22.8	
	Five		7.2		10.0	
	More than five		4.0		5.9	
Professional situation (%)				
	Retired	2.8		Out of the workforce		37.2
	Student	21.2				
	Unemployed	6.5		Unoccupied		6.6
	Public server	17.2		Occupied		39.1
	Worker – Own business	10.9				
	Worker – SME enterprises	15.4				
	Worker – Big enterprises	22.1				
	Other/No answer	3.9		Other		17.1

a Sample comprising 88.9% of the Brazilian states, 74.7% of the Brazilian capitals, and 4.7% of the Brazilian municipalities. More than 75% of the Brazilian municipalities are characterized as “small” (< 25,000 inhabitants), reducing the likelihood of achieving a substantial representativeness for them (31).

Our sample comprised 1,623 valid responses^b^, collected from almost all Brazilian states and capitals, but mainly from São Paulo (67%). While not representative of the Brazilian population, the study sample comes close to some sociodemographic characteristics, such as gender, age, residence area, number of households and professional situation^27^.

Beyond sociodemographic conditions, we also have collected data regarding political factors, organizational confidence, NPI compliance, perception of vaccine side effects and vaccine hesitancy (Appendix 1).

Respondents were asked to disclose their political leaning on a scale of 1 (Far Left) to 7 (Far Right) and to qualitatively evaluate (Very Bad, Bad, Good and Very Good) their perception of several institutions’ performance concerning the COVID-19 pandemic, including the Federal Government.

Regarding NPI compliance – mandatory mask use, social distancing (1,5 meters), respiratory etiquette, hand washing and staying at home –, respondents were asked to disclose their agreement level using a 5-point scale (disagree – agree), and their compliance level (never, rarely, frequently, and always) (Appendix 1). By means of a Principal Content Analysis (PCA), we used these questions to create a composite indicator labelled as “Compliance Index,” which represents 47.68% of the explanatory power of the total variables. Each measure contributed to the compliance index with different weights: mandatory mask use – 19.86%; social distancing (1.5 meters) – 21.84%; respiratory etiquette – 16.87%; hand washing – 20.49%; and staying at home, if possible – 20.94%.

As for vaccines, respondents were asked about their perception of vaccine side effects and willingness to be vaccinated (no, maybe, and yes).

### Data Analysis

We performed a set of bivariate analyses to understand the association between key variables – NPI Compliance Index, Age (years), Gender, Schooling level, Vaccine side effect, Political leaning and Government performance (Federal) – and willingness to take the COVID-19 vaccine. Subsequently, we used logistic regression models to estimate COVID-19 vaccine hesitancy. Using willingness to be vaccinated (measured by no/maybe (0), and yes (1)) as the dependent variable, the first model considers the baseline sociodemographic conditions as independent variables; the second model, in turn, includes political leaning, organizational confidence, non-pharmaceutical interventions compliance, and vaccine side effects as independent variables. Results are presented as Odds Ratios (OR), which indicate the odds of a dependent variable occurring in the presence or absence of the reference group, and as marginal effects (dy/dx), which tells us, in percentage points (pp), how a dependent variable changes when an explanatory variable changes, *ceteris paribus*.

## RESULTS

### Descriptive Statistics

Regarding the willingness to take the COVID-19 vaccine, 70% of the sample showed to be willing to take the COVID-19 shot, while almost 30% exhibited some degree of hesitancy (“not” or “maybe”) (Figure A). Such willingness to be vaccinated assumes that a vaccine is available for a given individual.

**Figure:**
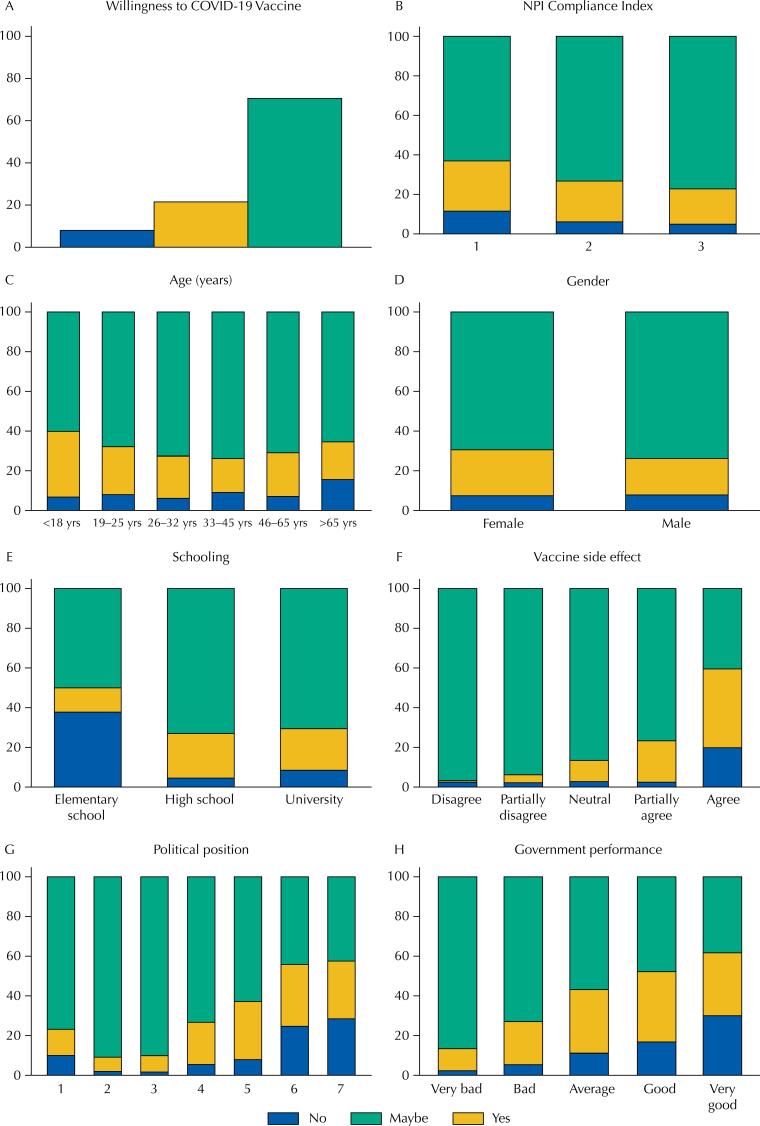
Bivariate analysis plots (except for 1A), respectively: Willingness to be vaccinated (A); Willingness to be vaccinated and NPI Compliance Index (B); Willingness to be vaccinated and Age (C); Willingness to be vaccinated and Gender (D); Willingness to be vaccinated and Schooling level (E); Willingness to be vaccinated and Vaccine side effects (F); Willingness to be vaccinated and Political leaning (G); Willingness to be vaccinated and Federal Government performance (H).

Plots 1B to 1H show the association between willingness to be vaccinated and the independent variables. Divided into tertiles, the NPI Compliance Index (Figure B) ranges from lower compliance (1) to higher compliance (3) levels, suggesting a possible association between this variable and willingness to be vaccine, with a higher percentage of “Yes” at the level “3”, than at the level “1.” Such findings may reflect the population’s level of concern: more concerned individuals are willing to be vaccinated and show higher compliance with sanitary measures.

As for the association between age and willingness to be vaccinated (Figure C), younger (less than 25 years) and older (more than 65 years) individuals showed higher levels of hesitancy, while those between 26 and 65 years old were less hesitant. In our sample, women showed greater hesitancy regarding the COVID-19 vaccine than men (Figure D).

As expected, the analysis found a strong association between schooling level and vaccine hesitancy (Figure E): individuals with only elementary schooling show vaccine hesitancy levels up to four times higher than those with higher schooling levels. Moreover, individuals more concerned with vaccine side effects show greater hesitancy in their willingness to be vaccinated (Figure F).

In our sample, right-wing individuals – generally more favorable to the current government – show higher levels of vaccine hesitancy than left-wing individuals. Together with the previous findings, this suggests that distrust in government is associated with higher compliance and vaccine acceptance, possibly due to high levels of concern (Figure G). We observed a similar inverse relationship between perception of government and willingness to be vaccinated (Figure H): respondents who scored government action as “Very bad” showed and 86% willingness to be vaccinated; among those who scored the government action as “Very good”, in turn, this willingness drops to 38%.

### Logistic Regression Models

In this study, we performed two regression models to estimate the factors associated with the willingness to take the COVID-19 vaccine. While model 1 includes only sociodemographic characteristics, model 2 considers the participants’ opinion on vaccine side effects, political leaning, perception of federal government performance and the compliance index^c^. This section focuses on the marginal effects analysis, but full results are shown below (Table 2).

**Table 2:** Logit models analyzing the explanatory capacity of the independent variables concerning the willingness to be vaccinated.

		(1)	(1)	(2)	(2)
		OR	dydx	OR	dydx
Compliance Index				1.123^b^	0.014^b^
Age (years)(baseline group ≤ 18)					
	19–25	0.912	-0.018	0.951	-0.006
	26–32	1.014	0.003	0.841	-0.020
	33–45	1.002	0.0004	0.877	-0.015
	46–64	0.785	-0.048	0.597	-0.063
	≥ 65	0.447	-0.174	0.619	-0.058
Gender (baseline group: Female)					
	Male	1.324^b^	0.054^b^	1.218	0.023
Professional situation (baseline group: Unemployed)					
	Retired	2.93^b^	0.179^c^	3.867^a^	0.145^b^
	Student	1.391	0.066	1.012	0.002
	Other	0.834	-0.039	1.080	0.010
	Public server	1.424	0.070	1.405	0.042
	Worker – Big enterprises	1.178	0.034	1.829	0.072
	Worker – SME enterprises	1.122	0.024	1.266	0.029
	Worker – Own business	0.936	-0.014	1.225	0.025
Schooling level (baseline group: Elementary school)					
	High school	2.940	0.246	2.856	0.127
	University – Bachelor	1.960	0.161	1.218	0.027
	University – MBAs and specializations	2.827	0.238	1.700	0.069
	University – Master	4.747 a	0.328	2.692	0.121
	University – PhD	5.103 a	0.338^∗^	2.049	0.091
Vaccine side effects (baseline group: do not disagree or agree)					
	Fully disagree			3.454 a	0.077^b^
	Partially disagree			2.346 a	0.060^b^
	Partially agree			0.503^b^	-0.077^c^
	Fully agree			0.108^c^	-0.344^c^
Political leaning (baseline group: Center)					
	1- Far left			0.896	-0.014
	2			1.869^b^	0.072^b^
	3			1.553 a	0.053 a
	5			0.690	-0.050
	6			0.476^c^	-0.104^b^
	7 - Far right			0.388^b^	-0.136^b^
Federal government - Performance (baseline group: Fair)					
	Very bad			2.355^c^	0.107^c^
	Bad			1.337	0.039
	Good			0.699	-0.052
	Very good			0.532	-0.095
	N	1,623	1,623	1,261	1,261

a, b, c indicate significance at 10%, 5% and 1% level, respectively.

Note: We also ran ordered logit models, which presented the same significative results.

In both models, age group does not seem to explain willingness to be vaccinated. Being retired is associated with the probability of taking the COVID-19 vaccine by 17.9 pp (p < 0.01) and by 14.5 pp (p < 0.05) in the first and second model, respectively, being the only professional situation with significant impact on the dependent variable – relative to being unemployed (baseline group). Although we found a positive impact associated with being male in the first model, this loses significance once we control for opinion on vaccine effects and compliance index. We observed similar results regarding educational variables such as Master’s and PhD programs.

The second model shows a negative and statistically significant association between fear of vaccine side effects and willingness to be vaccinated. Respondents who answered having no concern over vaccine side effects show higher levels of willingness to be vaccinated, with their probability changing by 7.7 pp (p < 0.10). On the other hand, individuals with high levels of concern about side effects have lower willingness to be vaccinated, varying by 34.4 pp (p < 0.01). Regarding political leaning, results show an association between being left-oriented and willingness to take the vaccine. Rating the government’s performance as “very bad” affects the probability of agreeing to be vaccinated by 10.7 pp (p < 0.01). The compliance index, which gives us an indicator of the participants’ overall level of compliance with all preventive measures, is in turn positively associated with willingness to vaccinate. An extra score on the compliance index means a 1.4 pp (p < 0.05) change in the probability of agreeing to vaccinate.

## DISCUSSION

This study investigated the association between social characteristics, political factors, and organizational performance and vaccine hesitancy in Brazil, contributing to understanding vaccine hesitancy factors in a LMIC context.

Our main finding suggests a negative association between positive perception of the federal government’s performance and willingness to be vaccinated, similar to previous studies on the likelihood of getting vaccinated in Brazil^26^. It also corroborates a North-American study, conducted during the Trump Administration, which suggested higher vaccine hesitancy among Trump supporters^18^. This phenomenon can be explained by the current Brazilian president’s negationist remarks regarding the COVID-19 pandemic and his position against compliance with NPIs and being vaccinated – a political scenario similar to the Trump administration^24,25,28^.

Regarding political leaning, our results show that espousing far-right ideology is positively associated with vaccine hesitancy, while being centre-left is associated with vaccine acceptance. This finding corroborates other studies on anti-vaccine movements and ideological isolation^11–13,26^) and reinforces the importance of political leadership in promoting compliance and public trust during crisis.

The NPI compliance index also provided interesting results, showing a positive association with willingness to be vaccinated. Such index is an innovative approach already used in previous studies^4,10,18^ and our results are in agreement with the literature^5,11,13^. We found a similar association regarding vaccine side effects, with more concerned individuals showing a positive association with willingness to be vaccinated. Such results highlight the importance of public communication about NPIs and vaccines.

This research has two major limitations. First, the method of data collection prevented us from obtaining a representative sample, particularly regarding the vulnerable population, which was underrepresented. Research shows that the most vulnerable individuals (with low schooling levels and high poverty levels) may express least willingness to be vaccinated^10,18–20^. If we transpose this scenario to the Brazilian context, then our vaccine hesitancy estimates should be interpreted as a lower bound. Like previous studies with convenient sampling methods^17,18^, however, the present study can still be used to derive significant policies. Even if the sample is not representative of the entire population, it can be for particular groups. Second, some respondents were not comfortable disclosing their political leanings, thus reducing the number of observations available in the second model. If such respondents are not distributed randomly, then the results may be biased.

Overall, the study contributes to a better understanding of vaccine hesitancy factors in a low-to-middle income country. Vaccine hesitancy is associated with multiple factors, such as NPIs compliance, sociodemographic and employment characteristics, political leaning, and public perception of government performance. Willingness to be vaccinated in Brazil is strongly associated with political leaning, perceived federal government performance, vaccine side effects, and compliance with non-pharmaceutical interventions. We found a strong association between vaccine hesitancy and being right-wing and positive perception of government performance. These findings suggest that the current distrust shown by the Brazilian president regarding vaccines contributes to vaccine hesitancy among his voter base. Individuals who oppose the current government, in turn, show higher willingness to be vaccinated.

## Footnotes

a Ministério da Saúde (BR), Conselho Nacional de Saúde. Resolução N° 466 de 12 de dezembro de 2012. Aprova diretrizes e normas regulamentadoras de pesquisas envolvendo seres humanos. Diário Oficial da União. 13 jun 2013; Seção 1: 59. Available from: https://conselho.saude.gov.br/resolucoes/2012/Reso466.pdf

b These responses include all completed and submitted responses recorded after validation testing. Participants were given the option not to disclose their political preferences, perception of vaccine side effects, perception of the federal government, and compliance levels.

c Compliance Index explained in detail in the methods section.
