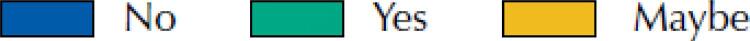# Errata

**DOI:** 10.11606/s1518-8787.2021055003903err

**Published:** 2022-01-11

In the article “**Running away from the jab: factors associated with COVID-19 vaccine hesitancy in Brazil**”, DOI https://doi.org/10.11606/s1518-8787.2021055003903, published on the Revista de Saúde Pública. 2021;55:97, on page 5, in the Figure subtitle, where it reads:

**Figure:**
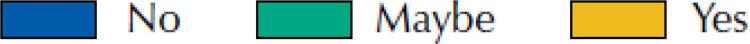

It should read as follows:

**Figure:**